# Mapping the Gene Ontology Into the Unified Medical Language System

**DOI:** 10.1002/cfg.407

**Published:** 2004-06

**Authors:** Jane Lomax, Alexa T. McCray

**Affiliations:** 1 European Bioinformatics Institute Hinxton Cambridge CB10 1SD UK; 2 National Library of Medicine Bethesda MD USA

## Abstract

We have recently mapped the Gene Ontology (GO), developed by the Gene Ontology Consortium, into the National Library of Medicine's Unified Medical Language System (UMLS). GO has been developed for the purpose of annotating gene products in genome databases, and the UMLS has been developed as a framework for integrating large numbers of disparate terminologies, primarily for the purpose of providing better access to biomedical information sources. The mapping of GO to UMLS highlighted issues in both terminology systems. After some initial explorations and discussions between the UMLS and GO teams, the GO was integrated with the UMLS. Overall, a total of 23% of the GO terms either matched directly (3%) or linked (20%) to existing UMLS concepts. All GO terms now have a corresponding, official UMLS concept, and the entire vocabulary is available through the web-based UMLS Knowledge Source Server. The mapping of the Gene Ontology, with its focus on structures, processes and functions at the molecular level, to the existing broad coverage UMLS should contribute to linking the language and practices of clinical medicine to the language and practices of genomics.


**Comparative and Functional Genomics**
*Comp Funct Genom* 2004; **5**: 354–361.
Published online in Wiley InterScience (www.interscience.wiley.com). **DOI:** 10.1002/cfg.407

**Research Paper**

### Mapping the Gene Ontology into the Unified Medical Language System

Jane Lomax[1]* and Alexa T. McCray[2]
[1] *European Bioinformatics Institute, Hinxton, Cambridge CB10 1SD, UK*
[2] *National Library of Medicine, Bethesda, MD, USA*

**Correspondence to: Jane Lomax, European Bioinformatics Institute, Hinxton, Cambridge CB10 1SD, UK. E-mail: jane@ebi.ac.uk*

Received: 5 August 2003
Revised: 9 March 2004
Accepted: 16 March 2004

### Abstract

**We have recently mapped the Gene Ontology (GO), developed by the Gene Ontology Consortium, into the National Library of Medicine's Unified Medical Language System (UMLS). GO has been developed for the purpose of annotating gene products in genome databases, and the UMLS has been developed as a framework for integrating large numbers of disparate terminologies, primarily for the purpose of providing better access to biomedical information sources. The mapping of GO to UMLS highlighted issues in both terminology systems. After some initial explorations and discussions between the UMLS and GO teams, the GO was integrated with the UMLS. Overall, a total of 23% of the GO terms either matched directly (3%) or linked (20%) to existing UMLS concepts. All GO terms now have a corresponding, official UMLS concept, and the entire vocabulary is available through the web-based UMLS Knowledge Source Server. The mapping of the Gene Ontology, with its focus on structures, processes and functions at the molecular level, to the existing broad coverage UMLS should contribute to linking the language and practices of clinical medicine to the language and practices of genomics. Copyright © 2004 John Wiley & Sons, Ltd.**

**Keywords:** **ontologies; semantic networks; controlled vocabularies; Gene Ontology; Unified Medical Language System**

### Introduction

A significant number of databases are now collecting and cataloguing genomic data. An annual review of molecular biology databases, for example, lists several hundred databases relevant to the genomic domain (Baxevanis, 2003). The databases that will most likely have the greatest impact are those that are able to link transparently to other closely related resources. One way to ensure this transparency is to interrelate the terminologies that are used by these various resources, be they databases of DNA sequences, literature databases or medical record systems (Harris and Parkinson, 2003). To this end we have recently mapped the Gene Ontology into the Unified Medical Language System. Ongoing and rapid advances in our understanding of genomic phenomena are becoming increasingly important to clinical research and clinical medicine (Ansell *et al.*, 2003; Cooper and Psaty, 2003; Collins, 1999). Mapping the large Gene Ontology, with its focus on structures, processes and functions at the molecular level, into the existing broad coverage UMLS should contribute to linking the language and practices of clinical medicine to the language and practices of genomics.

### The Gene Ontology

The Gene Ontology (GO) is a shared resource developed by the Gene Ontology Consortium, a group of researchers working on various model organism gene and protein databases. The primary use of GO is to produce functional annotations

Copyright © 2004 John Wiley & Sons, Ltd.

of genes and gene products in these databases, but it has found many other applications in areas such as microarray analysis, natural language processing and prediction of gene function. GO is a set of hierarchical, controlled vocabularies that describe certain biological phenomena, structured as directed acyclic graphs (DAGs), meaning that terms can have multiple parentage. There are three orthogonal vocabularies; biological process, molecular function and cellular component. Molecular functions are activities performed at the molecular level, while biological processes represent ordered assemblies of molecular functions. Cellular components are cellular locations, which include macromolecular complexes. GO is non-species-specific, thereby allowing cross-species comparison of entities in different databases.

GO is a dynamic vocabulary, which is constantly evolving and being updated in response to requests from the research community. These requests are managed via a publicly accessible website tracker (SourceForge GO Curator Requests Tracker, **https://sourceforge.net/tracker/?func=browse&group_id=36855&atid=440764**). Members of the consortium also organize and develop specific areas of the ontologies according to their specialist knowledge. There are several full-time editors who manage updates and who ensure the integrity of the overall resource. GO evolves by consensus, with any major changes in content or philosophy being discussed at regular meetings of the GO Consortium and on various e-mail lists. GO is available in different formats: flat files, which are the primary format, XML, and also as a MySQL database. The gene ontology and its annotations (gene associations) are freely available, with no licensing requirements.

Underlying GO are various philosophies and guidelines that are more fully documented elsewhere (Gene Ontology Consortium, 2000, 2001), but there are some assumptions that are important to discuss here. The first is the 'true path rule'. This rule states that the path from each term, back up through the hierarchy to its highest level parent, must be biologically accurate. For example, 'chitin biosynthesis' cannot be a descendant of 'cell wall biosynthesis' because chitin is also synthesized in the production of other structures, e.g. a cuticle (Hill *et al.*, 2002). Second, the scope of vocabularies is restricted, such that they do not include instances of individual gene products. Molecular function terms all represent activities or actions, rather than entities. Cellular component terms only represent locations, which include multigene–product complexes such as 'origin recognition complex'. Third, there are two different relationships a GO term can have to its parent, 'is_a' and 'part_of'. In the biological process ontology, 'part_of' refers to a sub-process, while in the cellular component ontology it refers to a sub-component.

### The Unified Medical Language System

The UMLS is a set of knowledge sources developed at the U.S. National Library of Medicine (Lindberg *et al.*, 1993; Unified Medical Language System; **http://umlsinfo.nlm.nih.gov/**). The UMLS aims to provide integrated access to a large number of biomedical information resources by unifying the vocabularies that are used to access those resources. The UMLS is currently used in a broad range of biomedical applications, including systems focused on patient data, digital libraries, Web and bibliographic retrieval, and medical decision support. Research groups use the UMLS to investigate a variety of natural language processing, knowledge representation and information retrieval questions. The UMLS consists of three knowledge sources; the Metathesaurus, the UMLS semantic network, and the Specialist lexicon with its associated lexical tools. The Metathesaurus contains information about biomedical concepts and consists of a variety of terminology systems, including thesauri, classification schemes, coding systems, and lists of controlled terms that have been independently developed by a broad range of different groups and organizations. These include medical specialty associations, hospital system developers, national and international standards bodies, informatics researchers, government agencies, and other health organizations. The Metathesaurus currently interrelates more than 60 families of vocabularies and consists of over 900 000 concepts.

Each Metathesaurus concept is assigned one or more semantic types from the UMLS semantic network. The network consists of 135 semantic types and 54 relationships, including 'is_a' and 'part_of', but also many other relationships important for the biomedical domain. These include five categories

Copyright © 2004 John Wiley & Sons, Ltd. 

of relationships, physical, functional, spatial, temporal, and conceptual. The semantic network provides a coherent framework for the sometimes quite disparate vocabularies that comprise the Metathesaurus (McCray, 2003). The 'true path rule' that has been described for the Gene Ontology applies to the UMLS semantic network, but it does not apply to the Metathesaurus itself. The terminologies are interrelated at the individual concept level, with many concepts consisting of synonyms drawn from a variety of terminologies, but there is no attempt to fully merge the hierarchical structures of the constituent terminologies (for discussion of the issues involved in mapping, merging, and aligning existing terminologies and ontologies, see Tuttle *et al*., 1995; Zeng and Cimino, 1996; Reed and Lenat, 2002; Pinto *et al*., 1999, Oliver *et al*., 1999). The Specialist lexicon and related lexical tools are resources that are used to manage lexical variation in a range of natural language processing applications (McCray *et al*., 1994).

The UMLS has been updated regularly since its first release in 1990. Until recently these were annual releases, but since 2002 they have been quarterly releases in response to requests from the active UMLS user community. Because some of the constituent vocabularies in the UMLS are governed by varying levels of copyright restriction, users are asked to sign a license agreement before accessing the UMLS data. There is no charge involved. With each release the knowledge sources are enhanced with both additional content and additional tools. Additions and enhancements to the UMLS are accomplished through a combination of automated and semi-automated processes, and a variety of tools have been developed to assist in the extensive human review process that follows the incorporation of any new UMLS content.

The UMLS knowledge sources are available as a set of ASCII relational tables and are fully accessible through the UMLS Knowledge Source Server, either through its Web interface or through its application programming interface, which gives the results as XML objects (Bangalore *et al*., 2003). The Knowledge Source Server retrieves information about particular concepts, including attributes such as the concept's definition, its semantic types, and the concepts that are related to it. It is also possible to limit a query to the perspective of a particular constituent vocabulary. For example, the user may be interested in seeing the ancestors or descendants for a term in just one particular vocabulary, or may like to know which synonyms originated in that specific vocabulary.

### Methods

Mapping the Gene Ontology into the UMLS involved several initial explorations before the final integration stage. First, we used automated procedures to determine whether there was any overlap between the coverage of GO and the coverage of the UMLS (McCray *et al*., 2002). Just over one-quarter of the GO terms were found in the UMLS. There was little overlap in biological processes (2%) and cellular components (3%), with the largest overlap in molecular functions (21%). However, later investigations showed that the apparent large overlap in the latter category resulted from certain assumptions about the nature of the molecular function terms in GO that needed to be reassessed.

Our second step involved a one month visit by the first author, who is a GO curator, to the National Library of Medicine to work with the UMLS team. The purpose of the visit was to ensure that each group would understand the goals, assumptions, and framework of the other group. We attempted to fully map GO into the UMLS during this time period, but we discovered a number of problems that needed to be addressed first. Thus, during this phase and in the subsequent months before the final mapping, many issues were addressed, debated and resolved, and these are discussed here.

The final mapping was done using the December 2002 version of GO. The GO at that time consisted of 6692 biological process terms, 5152 molecular functions and 1075 cellular components, for a total of 12 919 terms. We followed the same steps normally used to map a new vocabulary to the UMLS. First, the UMLS team studied the entire GO vocabulary and its documentation to assess its purpose, structure and explicit or implicit assumptions. In this case, the UMLS team was already well informed by extensive discussions with the GO team. Second, GO was examined for potential algorithmic assignments of UMLS semantic types. For example, the UMLS semantic type 'Cell Component' was a candidate for algorithmic assignment to all cellular component terms. Subsequent human

Copyright © 2004 John Wiley & Sons, Ltd. 

review in some cases altered these algorithmic assignments, but they were, nonetheless, a useful starting point for the reviewers. Next, the Specialist lexical programmes, together with other heuristics, were used to automatically map the terms in GO to existing concepts in the UMLS.

Finally, and importantly, each provisionally mapped concept was individually reviewed and sometimes modified by a UMLS editor. In some cases, an algorithmic mapping had proved incorrect, a particular mapping had been missed, or an incorrect semantic type had been assigned. UMLS editors review all aspects of a concept record, including not only semantic type assignments, but also definitions, related concepts (broader, narrower, and other related concepts), and a variety of other concept attributes.

### Results

The 2003AB version of the UMLS, available in July 2003, contains GO in its entirety. All GO terms now have a corresponding, official UMLS concept, and have therefore been assigned UMLS unique identifiers and semantic types from the UMLS semantic network. GO definitions and other GO term attributes have been incorporated and are available together with other UMLS concept attributes. GO terms are readily searchable through the UMLS Knowledge Source Server.

During the final mapping, only a small percentage of GO terms exactly matched existing UMLS concepts. This is perhaps not surprising, since GO is more specialized in the genomic domain than are any of the other vocabularies currently represented in the UMLS. Table 1 shows the results of mapping GO to the UMLS.

Overall, a total of 23% of the GO terms either matched directly (3%) or linked (20%) to existing UMLS concepts. Some examples of exact matches are 'DNA replication' (a GO biological process), 'extracellular matrix' (a GO cellular component), and 'protein binding' (a GO molecular function). Additionally, a number of relationships link GO terms to one or more existing UMLS concepts. Thus, a GO term might be narrower or broader than a UMLS concept, or some other relationship might obtain. For example, the GO term 'lipid metabolism' is narrower in meaning than the UMLS concept 'metabolism',

**Table 1.** The number of GO terms, by GO category, out of a total of 12 919 terms that either matched or linked to an existing UMLS concept

| | GO Category | | | |
|---|---|---|---|---|
| | **Molecular function** | **Biological process** | **Cellular component** | **Total** |
| Match to UMLS | 7 | 283 | 141 | 431 (3%) |
| Linked to UMLS | 2239 | 240 | 99 | 2578 (20%) |
| Total | 2246 | 523 | 240 | 3009 (23%) |

and the GO term 'feeding behaviour' is broader in meaning than the UMLS concept 'animal feeding behaviour'. GO terms may be related in other ways to UMLS concepts. There are many instances, for example, where the molecular activity of a particular enzyme, receptor, etc. is linked to the enzyme or receptor itself. Some examples are: 'T-cell receptor' (a UMLS concept) *exhibits* 'T-cell receptor activity' (a GO molecular function); and 'peroxisome assembly factor- 2′ (UMLS) *exhibits* 'peroxisome-assembly ATPase activity' (GO).

Some 45 of the 135 semantic types available in the UMLS semantic network were assigned to the GO terms. The vast majority were assigned to semantic types from the left hand side of the 'Biologic Function ' sub-tree, as shown in Figure 1.

In addition, many GO terms were assigned to the semantic type 'Cell Component' in the 'Anatomical Structure' sub-tree. Some terms were given the UMLS semantic types 'Individual Behaviour' (e.g. the GO term 'grooming behaviour') and 'Social Behaviour' (e.g. the GO term 'post-mating behaviour'). Some other semantic types that were assigned infrequently include 'Cell', 'Gene or Genome', and 'Body Space or Junction'. A total of 12 946 semantic types were assigned to the 12 919 GO terms. This means that in a few cases multiple semantic types were assigned to certain terms. An example is the GO term 'feeding behaviour', mentioned earlier, which was assigned to both 'Organism Function' and 'Individual Behaviour'.

Table 2 shows the most frequently assigned UMLS semantic types by GO category, and indicates that 99% (12 830/12 946) of all semantic types assigned fell into a total of nine UMLS semantic types.

Copyright © 2004 John Wiley & Sons, Ltd. 

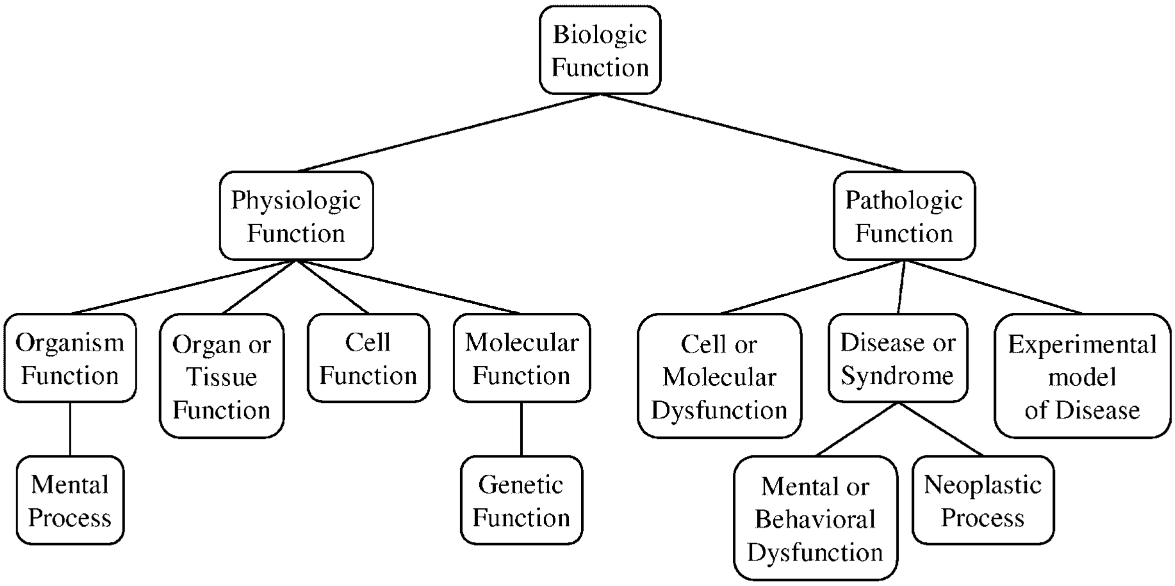

**Figure 1.** The 'Biologic Function' sub-tree in the UMLS Semantic Network

**Table 2.** The most frequent UMLS semantic types assigned to GO terms, by GO category

| UMLS semantic type | GO Category | | | |
|---|---|---|---|---|
| | Molecular function | Biological Process | Cellular component | Total |
| Biological function | | 12 | | 12 |
| Physiological function | 1 | 6 | | 7 |
| Organism function | | 416 | | 416 |
| Mental process | | 18 | | 18 |
| Organ or tissue function | | 352 | | 352 |
| Cell function | | 2311 | | 2311 |
| Molecular function | 4772 | 3211 | | 7983 |
| Genetic function | 375 | 302 | | 677 |
| Cell component | | 5 | 1049 | 1054 |
| Total | | | | 12 830 |

### Discussion

The mapping of GO to the UMLS highlighted issues in both terminology systems. The existing UMLS semantic types and their definitions caused some problems, and some naming issues in GO needed to be resolved. It has often proved to be the case that when new vocabularies are added to the UMLS, the developers of that vocabulary have seen opportunities to improve their terminology as a result of the mapping process; likewise, the UMLS developers have seen areas for improvement and enhancement.

In GO, a molecular function may be distinguished from a biological process by virtue of the fact that it is a direct activity, while a biological process is an ordered assembly of more than one activity. For example, the terms 'DNA binding' and 'DNA ligase' are molecular functions, while 'DNA repair' is a biological process. No similar distinction, however, is made within the UMLS semantic network. This means that a large proportion of both molecular function and biological process terms were assigned the same semantic type, 'Molecular Function' (or its child, 'Genetic Function'), thereby losing much of the resolution present in GO.

For some GO concepts, a precise semantic type was not available in the semantic network. For example, the UMLS semantic types 'Cell Function', and 'Molecular Function' are defined respectively as: 'A physiologic function inherent to cells or cell components'; and 'A physiologic function occurring at the molecular level'. The GO term 'cell cycle', for example, was assigned to 'Cell Function', but it was not entirely obvious which of the two semantic types to assign because this process occurs at both the molecular and whole cell level.

Copyright © 2004 John Wiley & Sons, Ltd. 

There are a relatively small number of semantic types at the level of molecular phenomena in the UMLS semantic network (see, however, Yu *et al.*, 1999, for some discussion of this point). For example, a comparison of the 'Biologically Active Substance' sub-tree of the UMLS semantic network with the 'Natural Phenomenon or Process' tree indicates that the former includes the semantic type 'Immunological Factor', for which there is, however, no corresponding 'Immunological Function' or 'Immunological Process'. This lack of resolution was also apparent in the semantic types assigned to GO cellular component terms, the great majority of which were given the semantic type 'Cell Component'. Additional semantic types that could be added as children of 'Cell Component' in the UMLS semantic network might include, for example, 'DNA component' and 'Membrane Component'. Some other GO categories that are not currently available as separate semantic types also became apparent, e.g. developmental processes. Thus, mapping GO to the UMLS raised a number of issues for the future development of the UMLS semantic network. In particular, the granularity of the semantic types available in the semantic network does not always allow for some of the finer distinctions that are made in GO. These possible areas for improvement are currently under discussion by the UMLS team.

Similarly, throughout the mapping process, several issues and areas for improvement within GO became apparent. For example, the GO molecular function tree has the major sub-tree 'enzyme', which describes catalytic activities (note that this sub-tree has subsequently been renamed 'catalytic activity'). The nomenclature of this tree often uses the names of the enzymes themselves so, for example, the GO term describing the catalytic activity of carbamate kinase is named simply 'carbamate kinase'. In biology, 'carbamate kinase' would commonly be used to refer to both the protein entity — the physical enzyme itself — and the activity of that enzyme. As a consequence, the GO term names were ambiguous, because it was not clear that they only represented the enzyme activity. This was not a problem within GO itself because this information is implicit; all enzyme terms are children of 'molecular function'. However, following the preliminary algorithmic mapping of GO to the UMLS (McCray *et al.*, 2002), we found that GO enzyme activity terms had been mapped into concepts representing protein entities because they shared identical text strings. In fact, we found that almost all GO molecular function term names were ambiguous, in that they shared a name with a protein entity, e.g. 'receptor', 'enzyme' and 'signal transducer'. To avoid multiple concepts with the same name and different meanings in the Metathesaurus, the word 'activity' was appended to all GO molecular function terms for the purposes of the mapping, with a few exceptions, including the term 'binding' and most of its children. Because of this change in GO naming, however, the exact number of matches of GO terms with existing UMLS concepts was significantly reduced from our earlier algorithmic results. The naming change does, however, better align the linguistic forms of the molecular function terms with their intended meanings. The naming change was agreed to at the January 2003 meeting of the entire GO Consortium and subsequently incorporated into GO itself.

In the molecular function tree, adding the word 'activity' to all terms made it clear that most represented genuine functions, e.g. 'receptor activity' and 'enzyme activity'. There were, however, still a few anomalies, e.g. 'structural molecule' terms actually result from a difficulty in describing the function of a gene product whose only 'activity' is to add to the architectural integrity of a structure, such as the mannoproteins that make up bacterial cell walls. These terms could be described as 'passive activities' and their existence in the GO molecular function tree, although at the moment necessary, is often debated. The exercise of adding 'activity' to molecular function term names in GO also helped highlight terms in GO that clearly didn't represent molecular activities, and many of these terms have subsequently been made obsolete.

We also encountered a problem with ambiguity in the GO cellular component tree. The locations in cellular components can be as granular as multi-subunit complexes, so in GO, the word 'complex' was added to the term name of all such structures to avoid cases of the same text string with different meanings. However, this addition still allows for ambiguity because a particular 'complex' can be used to describe a protein entity as well as a location, e.g. the 'origin recognition complex'. Again, to avoid creating concepts with the same name and different meanings, or

Copyright © 2004 John Wiley & Sons, Ltd. 

strings with different meanings within the same concept, the word 'location' was appended to all GO cellular component 'complex' terms within the UMLS. The original GO term is still preserved as a 'suppressible' form in the UMLS because, for some purposes, users may be interested in having access to the original GO strings. This appendage is only for the purposes of inclusion in the UMLS, so the original cellular component strings, without 'location', are still used in GO itself.

The preceding cases are examples of a common problem in vocabularies: that of the same text string with different meanings. In most cases these are ambiguities that have evolved in the language over time and that are only resolvable in their context of use. GO faces this problem in those cases where the same string may apply to different species, but with a different meaning in each. This arises because the terms in GO represent all of biology, covering species from viruses to human. An example of this is 'cell wall'. In plants, the 'cell wall' means the rigid membrane enclosing the protoplast of a cell, usually composed largely of cellulose, while in fungi, the same phrase is used to describe the structure surrounding the plasma membrane, usually composed of glycoproteins and peptidoglycans. There are undoubtedly similarities between these two structures, but they are by no means the same. To address this problem, GO uses the qualifier 'sensu', which is a taxonomic term meaning 'in the sense of'. In the case above, 'cell wall' appears in the GO cellular component ontology and has several children and grandchildren, including 'cell wall (sensu Fungi)' and 'cell wall (sensu Magnoliophyta)'. One child term has the meaning of a cell wall in the sense of fungi, and the other in the sense of flowering plants. The guide to whether new 'sensu' terms need to be created is whether or not they themselves will have different children. In this case, 'cell wall (sensu Fungi)' has the 'part of' child 'bud scar' while the plant term has a child term, 'cellulose microfibril'. This phenomenon also adds to the low exact match rate of GO terms to UMLS concepts, because concepts in the UMLS do not have species qualifiers.

Another issue that arose involved 'synonyms' in GO. Although called 'GO synonyms' for historical reasons, these text strings associated with GO terms frequently do not have an identical meaning to the main term, and are used mainly for the purposes of searching GO. Treating these as exact synonyms for the purposes of matching GO into the UMLS would have led to incorrect mappings, since each concept in the UMLS consists only of true synonyms. As a result, GO has been modified such that synonyms have now been labelled as to whether they are exact (true) synonyms, or whether they are broader, narrower, or otherwise related to the original GO term. These term–synonym relationships are currently stored as a separate file, but will soon be incorporated into GO itself.

### Conclusions

The work reported here highlights some of the issues that arise in mapping one terminology system into another. All terminology systems are developed with a particular purpose in mind, and this can have a significant impact on their design and implementation. The Gene Ontology was developed for the purpose of annotating gene products in genome databases, whereas the Unified Medical Language System was developed as a framework for integrating large numbers of disparate terminologies, primarily for the purpose of providing better access to biomedical information sources. The investigation revealed a variety of issues, some of them systematic, e.g. the nature of the GO molecular function terms, and some of them more idiosyncratic. The exact match rate of GO terms to existing UMLS concepts was low, which is most likely a reflection of the small number of existing UMLS vocabularies in the genomic domain. The UMLS semantic network, too, has a relatively small number of semantic types for representing this domain.

The mapping of GO into the UMLS should help improve interoperability among clinical, scientific literature and bioinformatics resources. Both GO and UMLS are dynamic systems that are used in a wide range of applications for a variety of research purposes. We will continue to collaborate on maintaining current versions of GO within UMLS, and we will investigate further methods for linking individual GO terms to existing UMLS concepts in order to effect the greatest integration possible. In addition, we will further investigate the UMLS semantic types and relationships for their usefulness in characterizing the genomic domain.

Copyright © 2004 John Wiley & Sons, Ltd.

### Acknowledgments

The authors wish to thank Bisharah Libbus, Stephanie Lipow, Stuart Nelson, Tammy Powell and Laura Roth of the UMLS team, and the GO Consortium, in particular Judith Blake, Michael Ashburner and Midori Harris, for their contributions to this effort.

### References

Ansell SM, Ackerman MJ, Black JL, Roberts LR, Tefferi A. 2003. Primer on medical genomics. Part VI: genomics and molecular genetics in clinical practice. *Mayo Clin Proc* **78**(3): 307–317.

Bangalore A, Thorn KE, Tilley C, Peters L. 2003. The UMLS Knowledge Source Server: An object model for delivering UMLS data. *Proc AMIA Symp* (in press).

Baxevanis AD. 2003. The Molecular Biology Database Collection: 2003 update. *Nucleic Acids Res* **31**(1): 1–12.

Collins FS. 1999. Genetics: an explosion of knowledge is transforming clinical practice. *Geriatrics* **54**(1): 41–47.

Cooper RS, Psaty BM. 2003. Genomics and medicine: distraction, incremental progress, or the dawn of a new age? *Ann Intern Med* **138**(7): 576–580.

Gene Ontology Consortium. 2000. Gene ontology: tool for the unification of biology. *Nature Genet* **25**(1): 25–29.

Harris MA, Parkinson H. 2003. Standards and ontologies for functional genomics: towards unified ontologies for biology and biomedicine. *Comp Funct Genom* **4**: 116–120.

Hill DP, Blake JA, Richardson JE, Ringwald M. 2002. Extension and integration of the gene ontology (GO): combining GO vocabularies with external vocabularies. *Genome Res* **12**(12): 1982–1991.

Lindberg DA, Humphreys BL, McCray AT. 1993. The Unified Medical Language System. *Methods Inf Med* **32**(4): 281–291.

McCray AT, Browne AC, Bodenreider O. 2002. The lexical properties of the gene ontology. *Proc American Medical Informatics Association (AMIA) Symp*, 504–508 (ISBN: 1-56053-600-4).

McCray AT, Srinivasan S, Browne AC. 1994. Lexical methods for managing variation in biomedical terminologies. *Proc Annu Symp Comput Appl Med Care* Hanley & Belfus: Washington DC; 235–239.

McCray AT. 2003. An upper level ontology for the biomedical domain. *Comp Funct Genom* **4**: 80–84.

Oliver DE, Shahar Y, Shortliffe EH, Musen MA. 1999. Representation of change in controlled medical terminologies. *Artif Intell Med* **15**(1): 53–76.

Pinto HS, Gomez-Perez A, Martins JP. 1999. Some issues on ontology integration. Proc IJCAI-99. Workshop on ontologies and problem-solving methods: **http://citeseer.nj.nec.com/pinto99 some.html**.

Reed SL, Lenat D. 2002. Mapping ontologies into Cyc. *Proc AAAI 2002*, AAAI 2002 Conference Workshop on Ontologies for the semantic web, Edmonton, Canada. **http;//citeseer.nj.nec.com/509238.html**.

SourceForge GO Curator Requests Tracker. **https://source forge.net/tracker/?func=browse&group_id=36855&atid=440764**.

The Gene Ontology Consortium. 2001. Creating the gene ontology resource: design and implementation. *Genome Res* **11**(8): 1425–1433.

Tuttle MS, Suarez-Munist ON, Olson NE, *et al.* 1995. Merging terminologies. *Medinfo* **8**(1): 162–166.

Unified Medical Language System: **http://umlsinfo.nlm.nih.gov/**.

Yu H, Friedman C, Rhzetsky A, Kra P. 1999. Representing genomic knowledge in the UMLS semantic network. *Proceedings of the AMIA Symposium*, 181–185 (ISBN: 1-56053-371-4).

Zeng Q, Cimino JJ. 1996. Mapping medical vocabularies to the Unified Medical Language System. *Proceedings of the AMIA Annual Fall Symposium*, 105–109.

Copyright © 2004 John Wiley & Sons, Ltd.
